# Supplementary material for: High and Hyper: Fentanyl Induces Psychomotor Side-Effects in Healthy Pigs
Source: Animals (Basel). 2023 May 17;13(10):1671. doi: 10.3390/ani13101671 (PMC10215380; doi:10.3390/ani13101671)
Supplement: Supplementary file 1 [file animals-13-01671-s001.zip › animals-2351808-supplementary.pdf]

Table S1. Repetitive behaviours (rooting behaviour, backward locomotion, circling, water nipple, and jumping) for each pig summarised for baseline, injection 1-3, and as a total sum. S-S: saline-saline group, F-S: fentanyl-saline group, F-K: fentanyl-ketanserin group.

**PIG**	**3**	**4**	**5**	**6**	**7**	**8**	**9**	**10**	**11**	**12**	**13**	**14**	**15**	**16**
Treatment group	**F-S**	**F-K**	**F-S**	**F-K**	**S-S**	**S-S**	**S-S**	**S-S**	**F-K**	**F-S**	**F-K**	**F-S**	**F-S**	**F-K**
**BASELINE**														
Sum of Rooting behaviour (Cumulative, s)	35	0	371	118	324	104	0	22	39	48	0	9	17	2
Sum of Backward (cumulative, s)	0	0	0	0	0	0	0	0	0	2	0	0	0	0
Sum of Circling (cumulative, s)	0	0	0	0	0	0	0	0	0	0	0	0	0	0
Sum of Water nipple (frequency)	1	0	0	0	1	0	0	1	1	0	0	0	1	0
Sum of Jumping (frequency)	0	0	0	0	1	1	0	0	1	0	0	0	0	0
**INJECTION 1**														
Sum of Rooting behaviour (Cumulative, s)	0	270	349	34	285	11	250	151	300	74	377	219	315	348
Sum of Backward (cumulative, s)	2	2	2	0	0	0	0	0	14	9	0	30	3	5
Sum of Circling (cumulative, s)	0	1	0	0	0	0	0	0	7	1	0	11	3	105
Sum of Water nipple (frequency)	21	3	0	5	1	0	1	0	5	5	0	5	1	0
Sum of Jumping (frequency)	2	0	0	34	0	1	0	0	0	0	0	0	0	0
**INJECTION 2**														
Sum of Rooting behaviour (Cumulative, s)	0	96	25	35	195	44	0	49	180	0	360	86	32	28
Sum of Backward (cumulative, s)	10	22	57	0	0	0	0	0	29	0	15	20	26	3
Sum of Circling (cumulative, s)	45	5	2	0	0	0	0	0	29	0	4	16	42	119
Sum of Water nipple (frequency)	29	0	0	0	0	1	0	0	13	0	0	0	0	0
Sum of Jumping (frequency)	13	0	0	24	0	0	0	0	7	24	0	0	0	0
**INJECTION 3**														
Sum of Rooting behaviour (Cumulative, s)	31	187	303	189	182	35	92	15	169	82	322	197	325	0
Sum of Backward (cumulative, s)	7	0	9	0	0	0	0	0	1	1	0	37	10	0
Sum of Circling (cumulative, s)	24	0	0	0	0	0	0	0	5	3	0	15	13	12
Sum of Water nipple (frequency)	18	2	1	1	0	1	0	0	7	15	1	0	1	0
Sum of Jumping (frequency)	31	0	0	3	0	0	0	0	16	2	2	0	0	0
**Total Sum of Rooting behaviour (Cumulative, s)**	**67**	**552**	**1049**	**377**	**986**	**194**	**342**	**237**	**688**	**204**	**1059**	**512**	**690**	**379**
**Total Sum of Backward (cumulative, s)**	**20**	**24**	**68**	**0**	**0**	**0**	**0**	**0**	**44**	**13**	**15**	**87**	**38**	**9**
**Total Sum of Circling (cumulative, s)**	**69**	**6**	**2**	**0**	**0**	**0**	**0**	**0**	**41**	**4**	**4**	**42**	**58**	**236**
**Total Sum of Water nipple (frequency)**	**69**	**5**	**1**	**6**	**2**	**2**	**1**	**1**	**26**	**20**	**1**	**5**	**3**	**0**
**Total Sum of Jumping (frequency)**	**46**	**0**	**0**	**61**	**1**	**2**	**0**	**0**	**24**	**26**	**2**	**0**	**0**	**0**

Table S2. Median (range) frequency or duration of the different behaviours for each treatment groups. i.: Saline-saline: saline injection 1-4. ii.Fentanyl: joined variables from the fentanyl-ketanserin and fentanyl-saline groups for baseline, injection 1 and injection 2. Variables from injection 3 and injection 4 is subdivided into the two different groups iia = fentanyl-ketanserin, and iib = fentanyl-saline. sec = seconds.

Behaviour per 10 minutes	Baseline	Injection 1	Injection 2	Injection 3		Injection 4	
Play (Duration, sec)							
i.*Saline*	0 (0-80)	1 (0-126)	2 (0-38)	*i.saline*	20 (0-51)	*i. saline*	8 (0-47)
ii.*Fentanyl*	0 (0-63)	0 (0)	0 (0)	*iia. ketanserin*	0 (0)	*iia. saline*	0 (0)
				iib. saline	0 (0)	*iib. ketanserin*	0 (0)
Sternal recumbency (duration, sec)							
i.*Saline*	190 (27-599)	234 (99-362)	331(258-509)	*i. saline*	350 (333-373)	*i. saline*	205 (96-521)
ii.*Fentanyl*	375 (74-548)	0 (0-3)	0 (0)	*iia. ketanserin*	0 (0-510)	*iia. saline*	0 (0-6)
				iib. saline	0 (0-70)	*iib. ketanserin*	0 (0-178)
Lateral recumbency (duration, sec)							
i.*Saline*	9 (0-485)	0 (0-115)	0 (22-67)	*i. saline*	41 (6-128)	*i. saline*	8 (0-58)
ii.*Fentanyl*	2 (0-298)	0 (0)	0 (0)	*iia. ketanserin*	0 (0)	*iia. saline*	0 (0)
				iib. saline	0 (0)	*iib. ketanserin*	0 (0)
Stiff gait (Duration, sec)							
i.*Saline*	0 (0)	0 (0)	0 (0)	*i. saline*	0 (0)	*i. saline*	0 (0)
ii.*Fentanyl*	0 (0)	187 (56-393)	300 (165-362)	*iia. ketanserin*	0 (0-4)	*iia. saline*	0 (0)
				iib. saline	125 (96-338)	*iib. ketanserin*	0 (0)
Ataxic gait (Duration, sec)							
i.*Saline*	0 (0)	0 (0)	0 (0)	*i. saline*	0 (0)	*i. saline*	0 (0)
ii.*Fentanyl*	0 (0)	0 (0)	0 (0)	*iia. ketanserin*	20 (0-74)	*iia. ketanserin*	0 (0-11)
				iib. saline	0 (0)	*iib. saline*	180 (18-334)
Freeze (frequency)							
i.*Saline*	0 (0)	(0)	(0)	*i. saline*	0 (0)	*i. saline*	0 (0)
ii.*Fentanyl*	0 (0)	41 (3-62)	82 (13-104)	*iia. ketanserin*	1 (0-11)	*iia. saline*	0 (0-1)
				iib. saline	29 (24-61)	*iib. ketanserin*	0 (0)
Water nipple (frequency)							
i.*Saline*	0 (0-0.5)	0 (0-1)	0 (0-1)	*i. saline*	0 (0-1)	*i. saline*	0 (0-1)
ii.*Fentanyl*	0 (0-2)	4 (0-21)	0 (0-29)	*iia. ketanserin*	1 (0-7)	*iia. saline*	1 (0-5)
				iib. saline	1 (0-18)	*iib. ketanserin*	1 (0-4)
Jumping (frequency)							
i.*Saline*	0 (0-1)	0 (0-1)	0 (0)	*i. saline*	0 (0)	*i. saline*	0 (0-3)
ii.*Fentanyl*	0 (0-2)	0 (0-34)	0 (0-24)	*iia. ketanserin*	2 (0-16)	*iia. saline*	5 (0-21)
				iib. saline	0 (0-31)	*iib. ketanserin*	3 (0-6)
Backwards locomotion (Duration, sec)							
i.*Saline*	0 (0)	0 (0)	0 (0)	*i. saline*	0 (0)	*i. saline*	0 (0)
ii.*Fentanyl*	0 (0-5)	2 (0 -30)	18 (0-57)	*iia. ketanserin*	0 (0-1)	*iia. saline*	3 (0-24)
				iib. saline	9 (1-37)	*iib. ketanserin*	3 (0-7)
Circling							

(Duration, sec)							
i.*Saline*	0 (0)	0 (0)	0 (0)	*i. Saline*	0 (0)	*i. Saline*	0 (0)
ii.*Fentanyl*	0 (0)	1 (0-15)	4 (0-39)	*iia. ketanserin*	0 (0-12)	*iia. saline*	3 (0-12)
				iib. saline	13 (0-24)	*iib. ketanserin*	0 (0-16)
Rooting behaviour (duration, sec)							
iii.*Saline*	63 (0-324)	201 (11-285)	47 (0-195)	*i. saline*	64 (15-182)	*i. saline*	74 (0-211)
iv.*Fentanyl*	2 (0-371)	285 (0-377)	34 (0-360)	*iia. ketanserin*	187 (0-322)	*iia. saline*	286 (61-360)
				iib. saline	197 (31-325)	*iib. ketanserin*	214(129-326)
Sitting (duration, sec)							
v.*Saline*	0 (0-42)	15 (0-62)	39 (5-61)	*i. saline*	15 (2-25)	*i. saline*	0 (36-85)
vi.*Fentanyl*	6 (0-127)	0 (0-2)	0 (0-17)	*iia. ketanserin*	0 (0-54)	*iia. saline*	0 (0)
				iib. saline	5 (0-25)	*iib. ketanserin*	0 (0-125)
